# Evaluation of outcomes in the management of primary sporadic desmoid-type fibromatosis at a specialist soft tissue sarcoma unit

**DOI:** 10.1186/s40001-022-00751-7

**Published:** 2022-07-16

**Authors:** Misbah Khan, Max Almond, Samuel Ford, Anant Desai

**Affiliations:** The Midland Abdominal and Retroperitoneal Sarcoma Unit (MARSU), University Hospitals Birmingham NHS Foundation Trust, Birmingham, B15 2GW UK

**Keywords:** Sporadic desmoid fibromatosis, Watchful waiting, Surgical resection of desmoid, Active surveillance, Prognostic factors, Event-free survival

## Abstract

**Background:**

Desmoids are rare fibroblastic tumours whose treatment in any individual case presents a persistent challenge. We endeavoured to evaluate various clinicopathological factors contributing to tumour behaviour.

**Methods:**

This is a retrospective review of 95 primary truncal sporadic fibromatosis managed between 2011 and 2020. We studied progression rate for wait and watch (WW) and recurrence rate for the surgically treated group as adverse events. Relevant event-free survivals and potential risk factors were analysed over a median follow-up of 27 months.

**Results:**

66 patients (69.5%) received watchful surveillance and 28 upfront surgery. 2-year progression-free survival in WW group (88.9%) was higher than RFS in the surgical group (77.1%) *p* = 0.02. Adverse event rate compared favourably, 28.8% in WW and 28.6% in surgical group. At final follow-up, rate of stable disease for WW was 47%, and the regression rate was 24.2%. On Cox regression analysis, meantime to progress was 14 ± 2.0 months, with larger tumour size as a significant prognostic indicator (*p* = 0.05). Surgical group's mean time to recurrence was 13.8 ± 2.76 months, with tumour location a significant contributing factor (*p* = 0.05).

**Conclusions:**

This study confirms to the safety of both treatment approaches. Adverse event rates remained comparable, but event-free survival was longer for the watchful surveillance group.

## Background

Desmoids are rare proliferative fibroblastic tumours of deep soft tissues with potential for local infiltrative and aggressive growth but lacking the ability to metastasize [1, 2]. The disease has two main varieties: sporadic desmoid-type fibromatosis affecting mainly the abdominal wall, limbs or trunk and hereditary desmoids that tend to be multifocal, intra-abdominal and frequently aggressive in nature [3, 4]. The latter are associated with familial adenomatosis polyposis coli (FAP).

Sporadic tumours have a recognised unpredictable natural history. The spectrum ranges from those having an indolent clinical course or even spontaneous regression on a ‘wait and watch strategy’, to rapidly growing locally aggressive tumours [5]. Some of these will develop local recurrence after complete surgical resection or require adjuvant treatment modalities for control of the disease [6]. Pain is the most common and debilitating symptom of the disease and is a potential side effect of treatment with a psychosocial impact that can be life-changing and lifelong [7, 8].

Although there has been a paradigm shift towards watchful waiting in past decades with commendable results [9, 10], it is this variable course of the disease combined with its relative rarity that still necessitates individualisation of management approach [11]. There has also been a progressive increase in the understanding of the disease with recent efforts at consolidating, mostly retrospective data, from various centres [12, 13]. Nevertheless, harmonising treatment strategies among clinicians at an international level requires additional pooling of data to look into various aspects of current practices, analyse outcomes, and define prognostic parameters [14, 15].

The objectives of this study were to evaluate and report upon the management of abdominal desmoid tumours at our centre and compare it with the national and international standards.

The primary endpoints were rate of progression and rate of regression for patients managed with the ‘Wait and watch’ approach, and rate of recurrence for the surgical resection group. Additional endpoints were the relevant event-free survivals and identification of potential variables correlated with disease aggressiveness in both groups.

## Methods

It is a retrospective analytic study design. The study was registered with the hospitals Clinical Audit & Registration management system (CARMS). Case identification was performed by filtration of The Midland Abdominal and Retroperitoneal Sarcoma Unit (MARSU) multidisciplinary team (MDT) meeting records for all diagnosed cases of truncal fibromatosis/desmoids referred to the centre between 1st March 2011 and 29th February 2020. The study was conducted in accordance with the good clinical practice and Declaration of Helsinki guidelines and work has been reported in line with the STROCSS criteria [16].

We searched the individual patient records of 133 diagnosed cases of desmoid-type fibromatosis. No limb tumours were part of the dataset. Patients with incomplete medical records and no follow-up with the department were excluded. Also excluded were cases with a recurrent disease on presentation and FAP-associated hereditary fibromatosis.

Date of last follow-up, treatment options utilised and tumour behaviour during the follow-up period was recorded on all patients. The median length of follow-up in our study was 27 months with an IQR of 10–47.

The active surveillance group included subjects observed with a wait and watch strategy (WW). Progression was defined as a failure of wait and watch approach by documentation of an increase in tumour size necessitating further intervention. Patient who underwent surgery were also evaluated for the development of recurrent disease. Throughout the study, surgical resections were performed by a set of trained sarcoma specialists. The disease course was analysed clinically as well as radiologically with all significant events including progression, stable disease, regression as well as recurrence documented in accordance with the RECIST [17].

Progressive disease on a WW approach or documented recurrence after surgical resection are scored as adverse events for stratification. Progression-free survival (PFS) was calculated from the time of first diagnostic imaging to evidence of clinical or radiological progression. Recurrence-free survival as the time from operation to either biopsy-proven or radiologic evidence of disease recurrence. The variables explored for a correlation with adverse events are age, hormonal association specifically related to pregnancy in females, tumour location and size, margin status in surgically resected patients and beta-catenin.

## Statistical analysis

Statistical analysis was performed using the IBM Statistical Package for the Social Sciences (SPSS) for Windows, Version 27.0. Mean values with standard deviation are used to describe continuous data, with discrete variables displayed as totals and frequencies. Continuous variables were dichotomised for imputation into uni- and multi-variate models. Binary and categorical comparisons are performed by utilising Chi-square test, while Cox’s proportional hazards regression model is used for multivariate modelling. Cumulative event rates calculation and survival curves are performed by utilising Cox regression analysis. A *P*-value of ≤ 0.05 is taken as a level of statistical significance.

## Results

Ninety-five patients with sporadic truncal desmoid-type fibromatosis were included in the final analysis. Clinical and demographic details of the patient population are shown in Table 1.

**Table 1:** Demographic and clinical characteristics of studied population

	*N* (%)	WW	Surgery
		*N* (%)	*N* (%)
Age at time of diagnosis	39.4	38.9	40.8(15.4;18–74)
years mean (SD; range)	(16.3; 16–90)	(16.7;13–90)	
Gender			
Male	30(31.6%)	17(25.8%)	12(42.9%)
Female	65(68.4%)	49(74.2%)	16(57.1%)
Hormonal association (pregnancy)			
Yes	29(30.5%)*	25(37.9%)	4(14.3%)
Tumour location			
Abdominal wall	47(49.5%)	40(60.6%)	7(25%)
Superficial trunk^**^	21(22.1%)	17(25.8%)	4(14.3%)
Intra-abdominal	26(27.4%)	9(13.6%)	17(60.7%)
Beta catenin on IHC			
+ ve	67(70.5%)	43(65.2%)	23(34.8%)
−ve	11(11.6%)	10(15.2%)	1(3.6%)
Incomplete/no record	17(17.9%)		
Tumour size			
< 5 cm	33(34.7%)	23(34.8%)	10(35.7%)
5-10 cm	47(49.5%)	35(53.0%)	11(39.3%)
> 10 cm	15(15.8%)	8(12.1%)	7(25%)

*IHC* immunohistochemistry

* *n* = 52 female patients of childbearing age

** Extra-abdominal trunk tumours included a wide range of locations including the neck, chest wall, axilla, breast and ischio-rectal fossa

69.5% of the patients (*n* = 66) were offered an initial period of active surveillance. The rest received treatment upfront, which essentially comprised surgery (*n* = 28, 29.5%). One patient received systemic treatment as a first-line and was excluded from the final analysis. Two-thirds of the total female and 57% of the male population received an upfront conservative approach. On a size-based criteria, more than 70% of small or intermediate-sized tumours and 50% of tumours larger than 10 cm were the ones who received an initial wait-and-watch approach.

Overall, the mean adverse event-free survival for the study group over the study period irrespective of the treatment offered was 154.6 ± 10.2 months. There were no disease-related deaths recorded over the study interval.

**Fig. 1:**
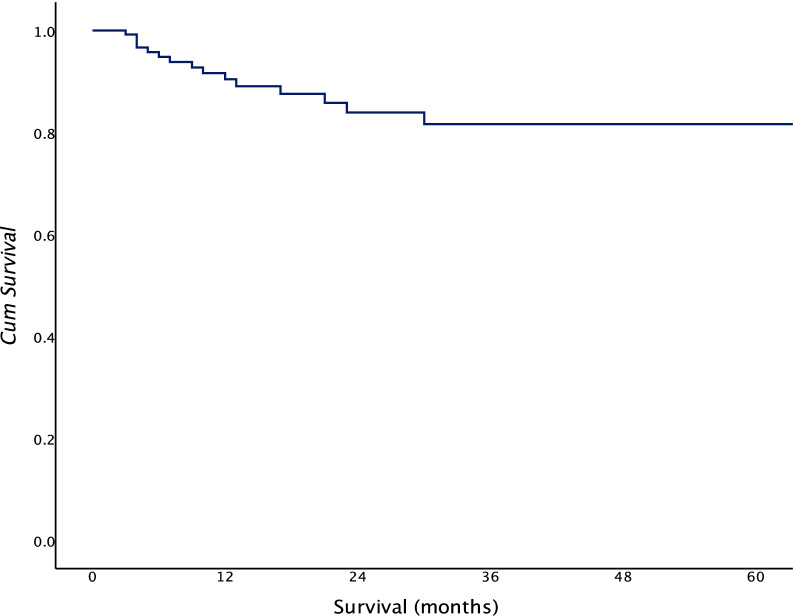
Overall adverse event-free survival

**Fig. 2:**
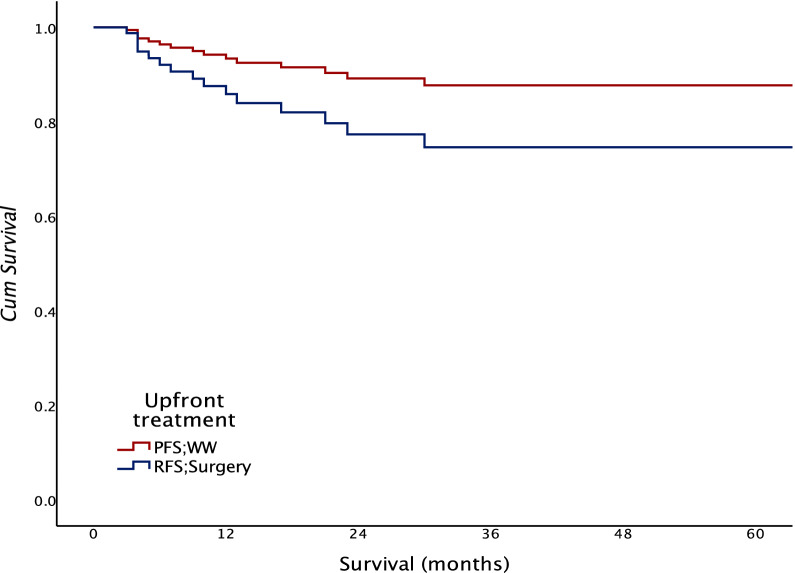
Adverse event-free survival according to the upfront treatment approach. ^*^PFS (progression-free survival), WW (watch and wait), RFS (recurrence-free survival)

Two- and 5-year adverse event-free survival was 83.8% and 81.94% for the whole group (Fig. 1). 2-year PFS was higher in the WW group at 88.9% than RFS in the surgical group at 77.1% (*p* = 0.02) (Fig. 2).

### Wait and watch

The rate of dimensionally stable disease from this population group over a median follow-up of 26 months was 47% (*n* = 31), regression was achieved in 24.2% (*n* = 16) at final follow-up, while the rate of progression was 28.8% (*n* = 19).

**Fig. 3:**
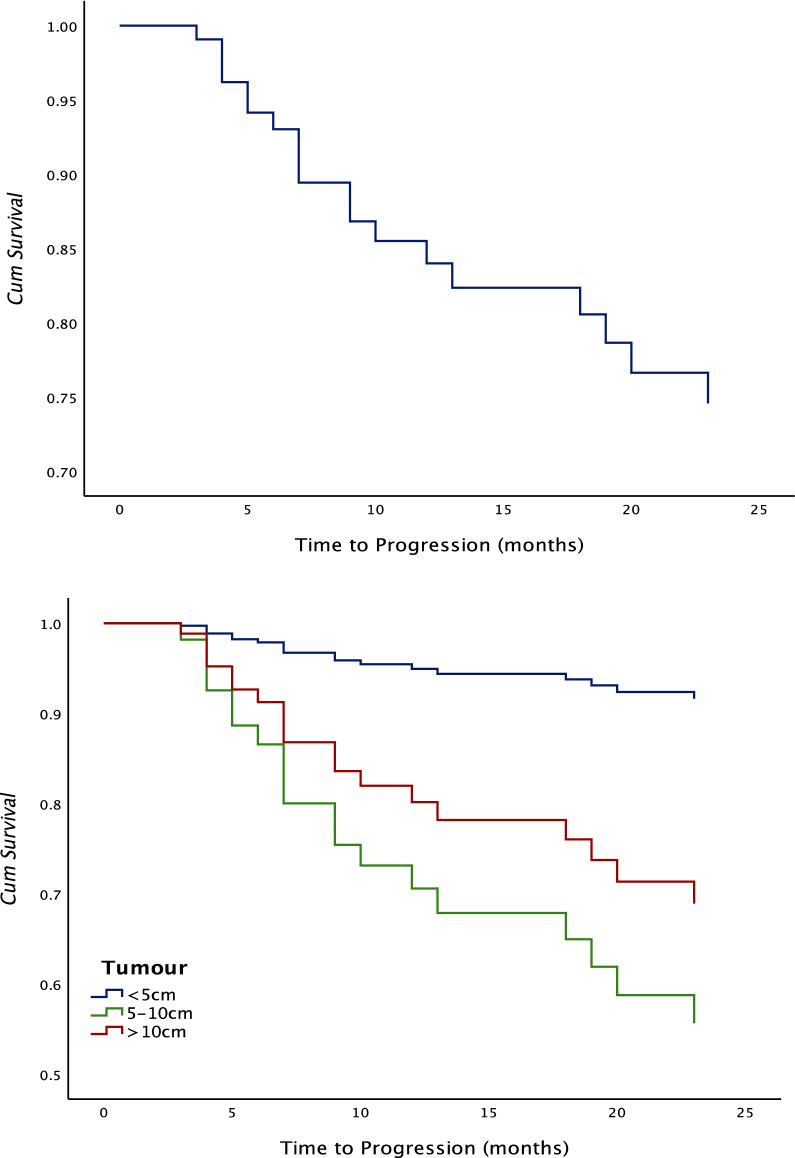
Time to progression: **a** overall, **b** according to tumour size

Mean time to progress was 14 ± 2.0 months (95% CI 7.22–12.75, Fig. 3). Of all the variables studied, tumour size at the time of initial presentation was the only positive predictor for progression (Table 2).

**Table 2:** Predicting factors for progression in patients managed with active surveillance

	*Univariate analysis*		*Multivariate analysis*		
	*p*-value	Odds ratio	*p*-value	Odds ratio	95% CI
*Age* < *50*	0.82	1.21	0.39	2.04	0.39–10.58
*Hormonal association*	0.05	4.2	0.53	1.51	0.42–5.45
*Tumour location*	0.39		0.38		
*Abdominal wall*	0.98	0.99	0.58	0.72	0.22–2.33
*Extra-abdominal Trunk*	0.99	0	0.17	0.21	0.02–1.92
*Beta catenin*					
+ *ve*	0.89	0.77	0.1	5.44	0.71–41.94
*Tumour size*	0.04		0.05		
*5-10 cm*	0.99	19.99	0.02	6.71	1.43–31.36
> *10 cm*	0.99	20.1	0.11	4.26	0.71–25.70

Reference categories are; age > 50 years, tumour location intra-abdominal, beta catenin −ve, tumour size < 5 cm

To achieve disease control, hormonal manipulation was provided to 12 and NSAIDs to 6 patients, with only 10 patients (15.1%) requiring further treatment. It included surgery in four, systemic chemotherapy in two and radiation therapy in one case, along with three patients (4.5%) who required complex and multiple lines of treatment, after an initial period of surveillance for a relentless disease course.

### Surgical outcomes

The total number of cases who had a surgical resection during their management were 35 (36.8%). Fourteen had localised tumour excision, and an additional four patients (12.8%) required some form of reconstruction, in addition to excision for their abdominal wall or trunk desmoids. Of the intra-abdominal tumours, two had simple tumour excision, with 15 having some form of visceral resection. Of all surgically resected specimens, 34.3% had a microscopically involved margin (R1), there were no R2 resections.

**Fig. 4:**
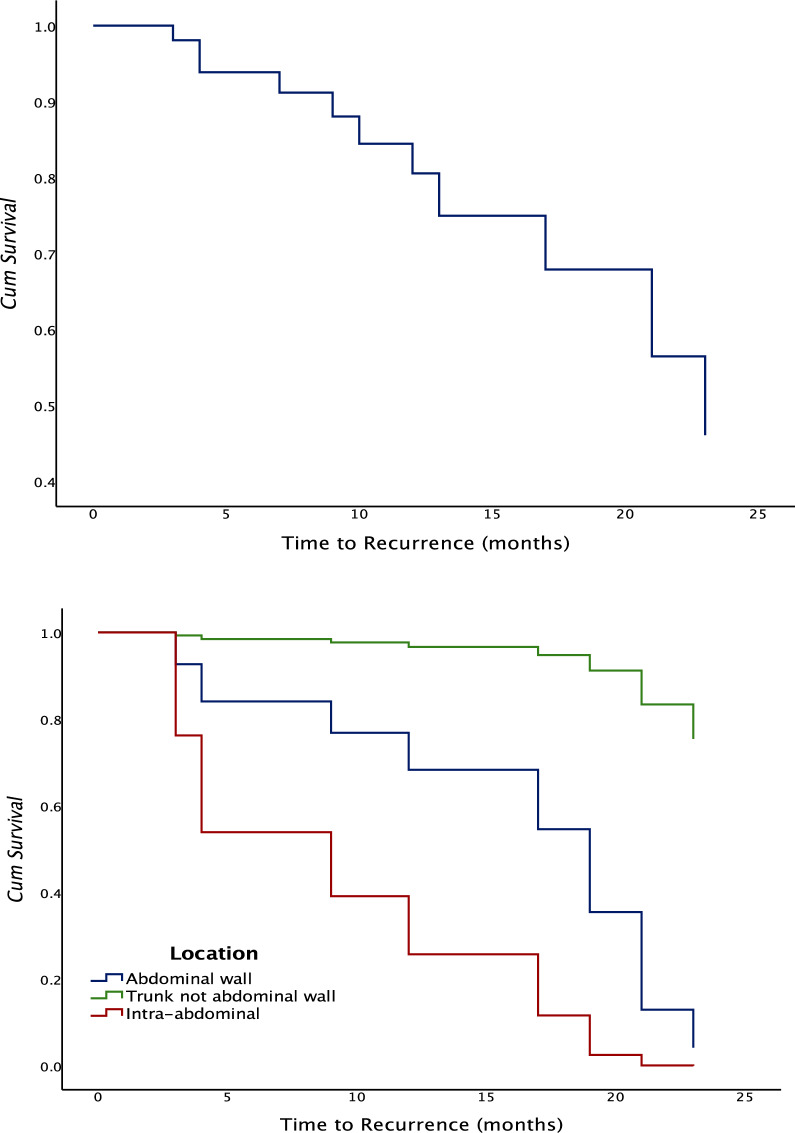
Time to recurrence: **a** overall, **b** according to tumour location

The rate of recurrence in the surgical group over a median follow-up of 30 months was 28.6% (*n* = 10), with a mean time to recurrence of 13.8 ± 2.76 SE of mean (95% CI 10.28–19.38). Most of the recurrences (92%) occurred within the first 24 months after surgery, with only one recorded event at 27 months (Fig. 4). In comparison with desmoids of the anterior abdominal wall, superficial trunk tumours with locations other than anterior abdominal wall were associated with better while intra-abdominal tumours with a worse recurrence-free survival (Table 3, Fig. 4).

**Table 3:** Predicting factors for recurrence in surgically managed subjects

	Univariate analysis		Multivariate analysis		
	*p*-value	Odds ratio	*p*-value	Hazard ratio	95% CI
*Age*					
< *50*	1	1	0.92	0.91	0.14–5.93
*Hormonal association*	0.78	0.71	0.32	2.55	0.40–16.03
*Tumour location*	0.97		0.05		
*Abdominal wall*	0.89	1.2	0.27	0.28	0.03–2.69
*Extra-abdominal Trunk*	0.95	0.92	0.02	0.03	0.00–0.51
*Beta catenin*					
+ *ve*	0.12	0.13	0.32	0.23	0.01–4.11
*Tumour size*	0.53		0.46		
*5-10 cm*	0.41	2.29	0.23	3.69	0.44–31.32
> *10 cm*	0.76	0.67	0.55	2.15	0.17–26.28
*Margins*					
+ *ve*	0.38	2.17	0.32	2.54	0.40–16.03
*Type of surgical resection*					
Complex^*^	0.85	1.15	0.27	0.36	0.06–2.19

Reference categories are; age > 50 years, tumour location intra-abdominal, beta catenin -ve, tumour size < 5 cm, margins –ve, type of surgical resection excision

^*^Multi-visceral resection for intra-abdominal/reconstruction for superficial tumours

## Discussion

Desmoid tumours are rare and have a well-recognised unpredictable natural history. To date, there remains insufficient and conflicting evidence to define any predictors of this varied behaviour. This translates into a persistent challenge in selecting the best treatment option for an individual patient. We, therefore, sought to evaluate various clinicopathological attributes in a series of primary sporadic desmoid patients managed at a single large soft tissue sarcoma unit.

In this study, we have excluded patients with hereditary fibromatosis, which is known to have an aggressive clinical course [18]. Also, to reduce the heterogeneity of the group, we have not included patients who presented with recurrent disease.

In recognition of the fact that a good proportion of desmoid tumours can regress spontaneously, an upfront wait and watch strategy is becoming the consensus first-line management approach [12, 13, 19]. In our study, 69.5% of patients were offered an initial trial of active surveillance. The rate of regression was 24.2%, while the rate of progression and 5-year progression-free survival was 28.8% and 87.8%, respectively. These results are consistent with and rather better than the internationally documented figures [20, 21]. Large tumour size on uni- and multi-variate regression analysis was found to be a significant factor with the tendency to progress over time. However, none of the other studied parameters, including association with pregnancy, were found to correlate on multivariate analysis. A recent study of 168 sporadic primary desmoids of all anatomical locations from a national high-volume centre showed a radiologic progression rate of 36% on active surveillance. The regression rate and rate of stable disease in their study was 27% and 36%, respectively [21]. This mirrors the results of our study as the population cohort is essentially similar other than the inclusion of limb fibromatosis cases in the French series. They also performed a regression analysis to find age younger than 50 years to be a significant contributor towards a higher progression rate (*p* = 0.046), which remains an insignificant contributor in our study group.

In the series by Van Houdt et al., treatment was offered to 46% (limb and trunk) and 43% of patients in our dataset [20]. The progression-free survival at 2 years (85.7%) was also comparable. The indication for active treatment in our study was morphologic progression with or without symptoms in 67.9% and symptomatic disease alone in 32.1%, which remains consistent with their results of 63% and 32%, respectively.

A systemic review of 211 patients from five centres reported an overall rate of progressive disease ranging from 4–34%, with a median time to progress between 14 and 20 months and time to regression of between 6 and 130 months. However, all progression events were recorded in the first 2 years after diagnosis. The studies included in this review have given widely varying results owing to the heterogeneous patient populations including both primary and recurrent disease as well as sporadic and hereditary fibromatosis. The study also included patients who had had previous surgery, a factor associated with increased risk of progression [22]. Mean time to progress in our study (14 ± 2 months) was nevertheless consistent with the above results. As symptomatic disease is the cause for a failed observation approach in about a third of patients, advancement in medical therapies for symptom control along with better psychological support to help patients could pave the way to superior outcomes [7, 8, 20, 23].

Several studies to date have analysed tumour recurrence after surgical resection. In a systemic review of 781 cases from 16 papers/studies, Smith et al. reported a local control rate of 42 to 86% for patients managed with surgery alone and a rate of 69 to 84% with the multimodality treatment. Their reported recurrence rate varied from 14 to 58% in included individual series [22]. Recurrence rate in our study after resection of primary disease was 28.6%, with a 2-year recurrence-free survival of 77.1% and a median time to relapse of 17 months. A large single-centre series of 495 surgically resected patients by Crago et al. reported a recurrence rate of 23% and 5-year recurrence-free survival of 69% [24]. On multivariate analysis, they found age (less than 26 years), large tumour size and tumour location (extremity and chest wall) associated with a shorter RFS. In another relatively recent series of 177 operated patients by Muller et al. [25], the observed recurrence rate over a median follow-up interval of 40 months was 29.4%, with a 5-year RFS of 61%. The study populations in both of the above series were fairly heterogeneous. Both datasets included hereditary fibromatosis, recurrent disease (23 and 25% of the total population in each series, respectively), adjuvant radiation treatment (18.7% and 20%), along the addition of systemic therapy in an additional addition subset in each. Nevertheless, summarising the above recent data with our results, documented recurrence rates remain less than 30% irrespective of the use of adjuvant or neo-adjuvant treatments. Microscopically involved tumour margin status in our surgically resected group failed to yield any correlation with the outcomes on both uni- and multi-variate analysis. Mullen et al., in their series found negative margin status (R0 resection), an independent prognostic factor with a better event-free survival on multivariate analysis. Nevertheless, the results may have been influenced by the addition of adjuvant treatment (29%), specifically radiation therapy in 20%. The recurrence rate for primary tumours subset in the study was 32%, with a median time to relapse of 14 months, results which are comparable to our study group [25]. Margin status (R1 vs R0) was not associated with altered outcome in the study cohort by Crago et al., but an R0 resection in the subgroup with smaller tumours < 5 cm was associated with a longer RFS (*p* = 0.007) [24]_._ Systemic review by Smith et al., has reported the R0 margin resection rate to a range between 18 to 100% [22]. Overall, margin status has been variably or poorly defined and remains a debatable predictor for determining recurrence. However, where possible, an R0 resection (margin > 1 mm), or at least a negative margin, is desirable [22, 24–27].

The overall adverse event-free survival has been described as better in the conservatively managed than the upfront surgery group (58 vs. 53% (*p* = 0.415)) [21]. Similar findings were noted in our study group with a statistically significant difference on Cox regression analysis for overall adverse event-free survival. Overall rate of event-free survival over 2 years in our study was 83.8%, which is much higher than 56% in the study by Penel et al [21]. Only a few cases had a relentless course in present study (5.3%) and required complex treatment strategies. That included, in addition to surgical resection, multiple chemotherapy courses, cryoablation in 2 patients, radiation therapy and RFA to one each.

As the management approach towards desmoids has evolved during the span of this study, more patients in the latter part of the study were offered active surveillance as a first-line treatment option. Also, the national guidelines for sarcoma patients have recently led to better referral pathways with more patients presenting upfront rather than after excision biopsy of these tumours in a local hospital [12]. Considering the above-mentioned selection biases and the inherent biases with a smaller subgroup population, we have not drawn a direct comparison analysis between the two management groups. However, the local control rate in both groups of primary sporadic truncal desmoids compared favourably (28.8% in W&W or 28.6% in the selective surgical group) with the previously reported series [20, 21]. Since the local control rate is very similar, an active surveillance approach in asymptomatic patients should be considered first-line, with patients that progress or become symptomatic being offered either medical management, or surgical resection aimed at achieving an R0 resection and preserving function [7, 11, 12].

The results from various single-centre series have failed consistently to correlate disease course and clinicopathological features. A collaborative effort with a larger homogenous dataset to elucidate the contribution of these on the clinical behaviour of this rare pathology and its management is imperative. Moreover, although we included beta-catenin staining on immunohistochemistry in our study, results of mutational analysis of the CTNNB1 gene were not studied, due to its relatively recent inclusion in clinical practice. Additional future perspectives also lie in identifying different molecular mechanisms to correlate with tumour biology and tumour behaviour in fibromatosis patients, including the role of different CTNNB1 subtypes. This may pave the way to developing stratified, and targeted treatment approaches towards tumours with potentially more aggressive behaviour [14, 27–29].

## Conclusions

The adverse event rate in the present study remained comparable for both treatment approaches and identical to the reported data. This confirms the safety of both treatment approaches in sporadic primary desmoids. However, the event-free survival in our study was better for the watchful surveillance group, which shall be given primary consideration. In addition, tumour size on initial surveillance and tumour location after complete surgical resection have been correlated with adverse event-free survivals in respective groups.

## Data Availability

Derived data supporting the findings of this study are available from the corresponding authors on a reasonable request.

## Abbreviations

W&W/WW: Wait and watch
FAP: Familial adenomatosis polyposis coli
CARMS: University hospitals Birmingham Clinical Audit & Registration management system
MARSU: Midland Abdominal and Retroperitoneal Sarcoma Unit
RECIST: Response evaluation criteria in solid tumours
IBM: International business machines
SPSS: Statistical Package for the Social Sciences
CI: Confidence interval
PFS: Progression-free survival
RFS: Regression-free survival
DFS: Disease-free survival
OS: Overall survival
IQR: Inter-quartile range
CTNNB1: Catenin beta-1
RFA: Radio-frequency ablation
NSAIDs: Non-steroidal anti-inflammatory drugs
